# Contribution of cardio-vascular risk factors to depressive status in the PREDIMED-PLUS Trial. A cross-sectional and a 2-year longitudinal study

**DOI:** 10.1371/journal.pone.0265079

**Published:** 2022-04-13

**Authors:** Sandra Martín-Peláez, Lluis Serra-Majem, Naomi Cano-Ibáñez, Miguel Ángel Martínez-González, Jordi Salas-Salvadó, Dolores Corella, Camille Lassale, Jose Alfredo Martínez, Ángel M. Alonso-Gómez, Julia Wärnberg, Jesús Vioque, Dora Romaguera, José López-Miranda, Ramón Estruch, Francisco J. Tinahones, José Lapetra, Fernando Fernández-Aranda, Aurora Bueno-Cavanillas, Josep A. Tur, Vicente Martín, Xavier Pintó, Miguel Delgado-Rodríguez, Pilar Matía, Josep Vidal, Clotilde Vázquez, Lidia Daimiel, Emili Ros, Estefanía Toledo, Stephanie K. Nishi, Jose V. Sorli, Mireia Malcampo, M. Ángeles Zulet, Anaí Moreno-Rodríguez, Raquel Cueto-Galán, Diego Vivancos-Aparicio, Antoni Colom, Antonio García-Ríos, Rosa Casas, M Rosa Bernal-López, Jose Manuel Santos-Lozano, Zenaida Vázquez, Carlos Gómez-Martínez, Carolina Ortega-Azorín, Jose Luís del Val, Itziar Abete, Amaia Goikoetxea-Bahon, Elena Pascual, Nerea Becerra-Tomás, Juan J. Chillarón, Almudena Sánchez-Villegas

**Affiliations:** 1 Department of Preventive Medicine and Public Health, University of Granada, Granada, Spain; 2 Instituto de Investigación Biosanitaria de Granada (ibs.GRANADA), Granada, Spain; 3 Centro de Investigación Biomédica en Red Fisiopatología de la Obesidad y la Nutrición (CIBEROBN), Institute of Health Carlos III, Madrid, Spain; 4 Research Institute of Biomedical and Health Sciences (IUIBS), University of Las Palmas de Gran Canaria, Las Palmas de Gran Canaria, Spain; 5 Centro de Investigación Biomédica en Red Epidemiología y Salud Pública (CIBERESP), Institute of Health Carlos III, Madrid, Spain; 6 Department of Preventive Medicine and Public Health, IdiSNA, University of Navarre, Pamplona, Spain; 7 Department of Nutrition, Harvard T.H. Chan School of Public Health, Boston, Massachusetts, United States of America; 8 Universitat Rovira i Virgili, Departament de Bioquímica i Biotecnologia, Unitat de Nutrició Humana. Reus, Spain; 9 University Hospital of Sant Joan de Reus, Nutrition Unit, Reus, Spain; 10 Institut d’Investigació Sanitària Pere Virgili (IISPV), Reus, Spain; 11 Department of Preventive Medicine, University of Valencia, Valencia, Spain; 12 Unit of Cardiovascular Risk and Nutrition, Institut Hospital del mar de Investigaciones Médicas Municipal d’Investigació Médica (IMIM), Barcelona, Spain; 13 Department of Nutrition, Food Sciences and Physiology, University of Navarra, Pamplona, Spain; 14 Precision Nutrition Program, IMDEA Food, CEI UAM + CSIC, Madrid, Spain; 15 Bioaraba Health Research Institute, Cardiovascular, Respiratory and Metabolic Area; Osakidetza Basque Health Service, Araba University Hospital; University of the Basque Country UPV/EHU, Vitoria-Gasteiz, Spain; 16 Department of Nursing, School of Health Sciences. University of Malaga- Instituto de Investigación Biomédica de Málaga (IBIMA), Málaga, Spain; 17 Instituto de Investigación Sanitaria y Biomédica de Alicante, ISABIAL-UMH. Alicante, Spain; 18 Health Research Institute of the Balearic Islands (IdISBa), Palma de Mallorca, Spain; 19 Lipids and Atherosclerosis Unit, Department of Internal Medicine, Maimonides Biomedical Research Institute of Córdoba (IMIBIC), Reina Sofía University Hospital, University of Córdoba, Córdoba, Spain; 20 Department of Internal Medicine, Institut dÌnvestigacions Biomèdiques August Pi Sunyer (IDIBAPS), Hospital Clinic, University of Barcelona, Barcelona, Spain; 21 Virgen de la Victoria Hospital, Department of Endocrinology, Instituto de Investigación Biomédica de Málaga (IBIMA). University of Málaga, Málaga, Spain; 22 Department of Family Medicine, Research Unit, Distrito Sanitario Atención Primaria Sevilla, Sevilla, Spain; 23 Research Group on Community Nutrition & Oxidative Stress, University of Balearic Islands, Palma de Mallorca, Spain; 24 Institute of Biomedicine (IBIOMED), University of León, León, Spain; 25 Lipids and Vascular Risk Unit, Internal Medicine, Hospital Universitario de Bellvitge, Hospitalet de Llobregat, Barcelona, Spain; 26 Center for Advanced Studies in Olive Grove and Olive Oils, University of Jaén, Jaén, Spain; 27 Department of Endocrinology and Nutrition, Instituto de Investigación Sanitaria Hospital Clínico San Carlos (IdISSC), Madrid, Spain; 28 CIBER Diabetes y Enfermedades Metabólicas (CIBERDEM), Instituto de Salud Carlos III (ISCIII), Madrid, Spain; 29 Department of Endocrinology, Institut dÌnvestigacions Biomèdiques August Pi Sunyer (IDIBAPS), Hospital Clinic, University of Barcelona, Barcelona, Spain; 30 Department of Endocrinology and Nutrition, Hospital Fundación Jiménez-Díaz, Instituto de Investigaciones Biomédicas IISFJD. University Autónoma, Madrid, Spain; 31 Nutritional Genomics and Epigenomics Group, IMDEA Food, CEI UAM + CSIC, Madrid, Spain; 32 Lipid Clinic, Department of Endocrinology and Nutrition, Institut d’Investigació Biomédiques August Pi Sunyer (IDIBAPS), Hospital Clinic, Barcelona, Spain; 33 Centro Salud San Vicente 1, Alicante, Spain; 34 Institute for Innovation & Sustainable Development in Food Chain. Universidad Pública de Navarra (UPNA), IdisNA, Pamplona, Spain; University of Malaya: Universiti Malaya, MALAYSIA

## Abstract

**Background:**

Cardio-vascular disease and depression are thought to be closely related, due to shared risk factors. The aim of the study was to determine the association between cardio-vascular risk (CVR) factors and depressive status in a population (55–75 years) with metabolic syndrome (MetS) from the PREDIMED-Plus trial.

**Methods and findings:**

Participants were classified into three groups of CVR according to the Framingham-based REGICOR function: (1) low (LR), (2) medium (MR) or (3) high/very high (HR). The Beck Depression Inventory-II (BDI-II) was used to assess depressive symptoms at baseline and after 2 years. The association between CVR and depressive status at baseline (n = 6545), and their changes after 2 years (n = 4566) were evaluated through multivariable regression models (logistic and linear models). HR women showed higher odds of depressive status than LR [OR (95% CI) = 1.78 (1.26, 2.50)]. MR and HR participants with total cholesterol <160 mg/mL showed higher odds of depression than LR [OR (95% CI) = 1.77 (1.13, 2.77) and 2.83 (1.25, 6.42) respectively)] but those with total cholesterol ≥280 mg/mL showed lower odds of depression than LR [OR (95% CI) = 0.26 (0.07, 0.98) and 0.23 (0.05, 0.95), respectively]. All participants decreased their BDI-II score after 2 years, being the decrease smaller in MR and HR diabetic compared to LR [adjusted mean±SE = -0.52±0.20, -0.41±0.27 and -1.25±0.31 respectively). MR and HR participants with total cholesterol between 240–279 mg/mL showed greater decreases in the BDI-II score compared to LR (adjusted mean±SE = -0.83±0.37, -0.77±0.64 and 0.97±0.52 respectively).

**Conclusions:**

Improving cardiovascular health could prevent the onset of depression in the elderly. Diabetes and total cholesterol in individuals at high CVR, may play a specific role in the precise response. International Standard Randomized Controlled Trial (ISRCTN89898870).

## Introduction

Cardiovascular disease (CVD) is the main cause of mortality worldwide [1], and increases dramatically with increasing age in both men and women. Additionally, there are clear male-female differences in cardiovascular system changes at older ages that may influence sex differences in CVD progression and outcome [2]. CVD and depression are thought to be closely related, due to similar risk factors, including inflammation and oxidative stress, among other factors, the key links amongst them [3]. Depression is the most prevalent of the mental illnesses, and it has been estimated to be the main cause of disability by 2030 [4]. Depressive disorders are more prevalent I women than in men, with a 2-fold increase in the probability of suffering from depression across a variety of nations, cultures, and ethnicities [5].

Although it has been shown that depression could be a risk factor for CVD [6,7], studies situating cardiovascular risk (CVR) as risk factor for developing depression are scarce [8]. Some studies have investigated cardiovascular health index on depression onset [9,10], in young adults [11] or in middle age populations. On the other hand, depressive disorders among CVD patients are still under-recognized and under-treated, particularly in women [12].

The association CVD and depression is thought to be mediated by the so-called metabolic syndrome (MetS). It has been suggested that prevention and treatment of MetS may be important for the prevention of depressive symptoms in middle aged and older adults aged 65 and 70 years [13]. In this sense, studies investigating the role of CVR factors in older adults suffering of MetS are needed to determine their relative importance on depression onset.

The objective of this study was to investigate the association between cardio-vascular risk (CVR) factors and depressive status in a population (55–75 years) with metabolic syndrome (MetS) from the PREDIMED-Plus trial.

## Methods

### Study design

In this sub-study of the PREDIMED-Plus trial, we analyzed the variables of interest as an observational longitudinal cohort. The PREDIMED-Plus study is an ongoing 6-year multicenter, randomized, parallel-group and primary prevention trial conducted in Spain. The aim of the trial is to assess the effect of an intensive weight loss intervention program based on an energy-restricted traditional Mediterranean diet, physical activity promotion behavioral support, on clinical cardiovascular events, compared to usual care and dietary counselling intervention only with energy unrestricted Mediterranean Diet (control group). The study protocol includes more detailed information and is available at the website https://www.predimedplus.com/ and in previous publications [14]. The database used in this study was updated on June 26^th^, 2020.

### Ethics approval

The study is conducted in accordance with the principles of the Declaration of Helsinki. The respective Institutional Review Board (IRB) of all study centers approved the study protocol. The trial was registered at the International Standard Randomized Controlled Trial in 2014 (ISRCTN89898870). All participants provided written informed consent.

### Participants and data collection procedures

**Fig 1:**
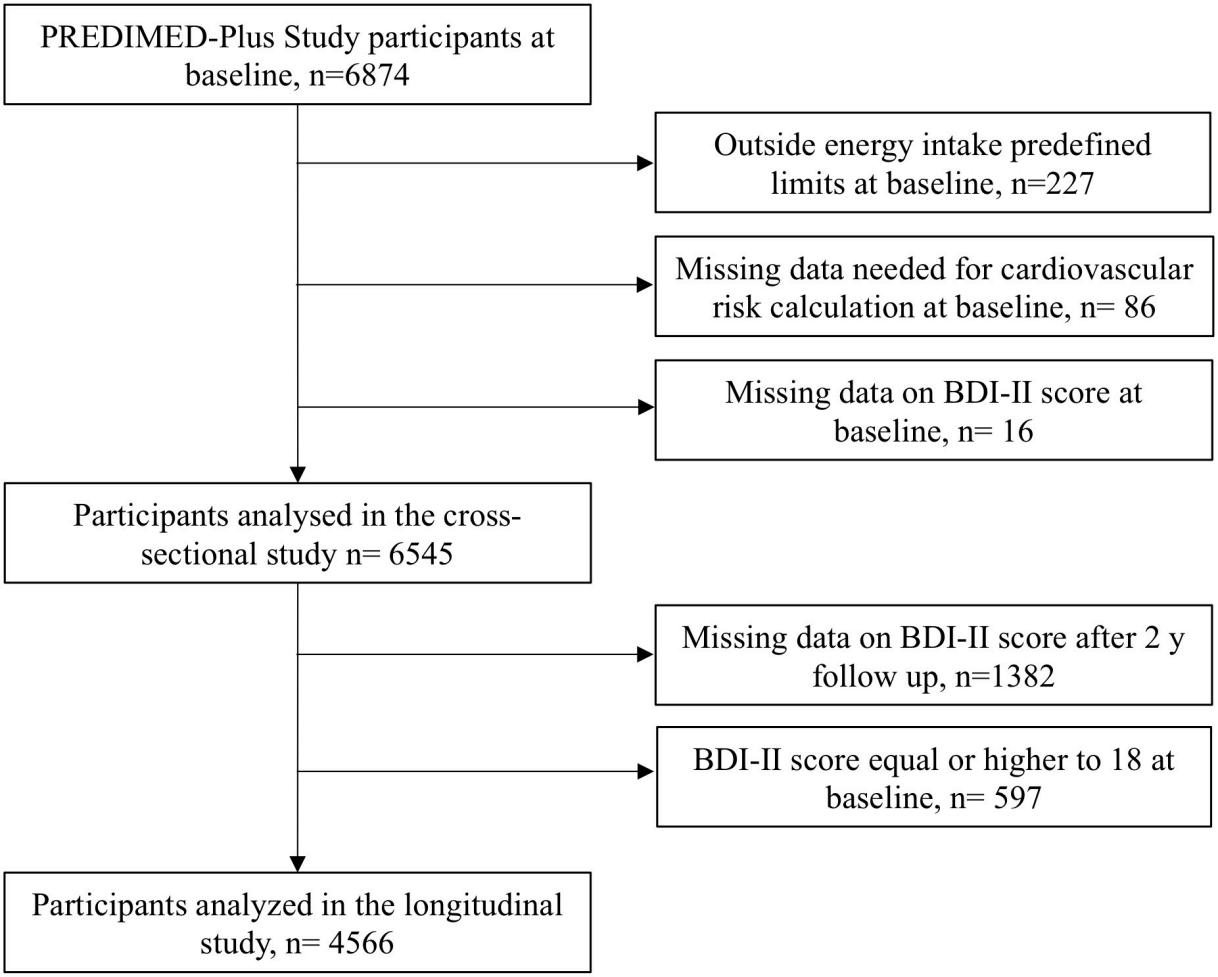
Flow chart for the analysis of cardiovascular risk and depression in the PREDIMED-PLUS trial. CVR, cardiovascular risk; BDI-II, Beck Depression Inventory-II.

The study participants were men (55–75 y) and women (60–75 y) with either overweight or obesity (body mass index (BMI) ≥27 kg/m^2^ or <40 kg/m^2^), suffering from MetS and not presenting at baseline neither cardiovascular disease, nor other neurological or endocrine diseases. From the 6874 participants enrolled, those lacking information about dietary intake or with values for total energy intake beyond predefined limits at baseline (<800 kcal/day or >4000 kcal/day for men; <500 kcal/day or >3500 kcal/day for women) [15] were excluded (n = 227). Participants lacking any of the variables needed for Framingham-based REGICOR CVR calculation (n = 86), and those who did not complete the Beck Depression Inventory-II (BDI-II) questionnaire at baseline (n = 16) were also excluded, remaining 6545 participants for the cross-sectional analysis. In addition, participants with no data for the BDI-II questionnaire after 2 years (n = 1382) and those whose BDI-II score was ≥18 at baseline (n = 597), were excluded, remaining 4566 participants for the longitudinal analysis (Fig 1).

### Cardiovascular risk calculation

Individual CVR was calculated from the Framingham-based REGICOR tables validated for the Spanish population [16]. These tables classify individual CVR into 4 categories (low, medium, high, and very high) according to age, sex, smoking habit, diabetes status, levels of total cholesterol, systolic blood pressure, and diastolic blood pressure. Individual CVR obtained from the tables was adjusted according to HDL-cholesterol values [16]. Afterwards, participants were divided into 3 groups of CVR: low (LR, CVR<5%), moderate (MR, CVR 5–9%) and high/very high (HR, CVR ≥10%). In addition, participants presenting at least one of the following circumstances: LDL-cholesterol≥240 mg/dL, SBP≥180 mm Hg and/or DBP≥110 mm Hg, or both, were included in the HR group [17].

### Outcome assessment

Trained PREDIMED-Plus staff collected individual information about depressive status using the BDI-II questionnaire previously validated in the Spanish population, at baseline and at 2 years of follow-up. BDI-II is composed by 21 questions, each of them with four possible answers sorted according to symptoms severity. Scores ranges from 0 to 63 points [18]. In the present analysis, our dependent variables were depressive status, BDI-II score and changes in depressive symptomatology. A depressive status (qualitative dichotomic variable) was considered when BDI-II score was of 18 or higher [19]. Changes in depressive symptomatology after 2 years of follow-up (quantitative variable) were obtained as the difference of the 2-year follow-up BDI-II score minus the baseline BDI-II score.

### Covariate assessment

At baseline and once yearly, trained staff collected information about socioeconomic and lifestyle factors including sex, age, body mass index (BMI), waist-hip ratio (WHR), educational level (primary school, secondary school, and higher education), civil status (married, divorced/separated, widow, single) whether they lived alone or not and employment status (active, retired, housewife, other). Other lifestyle variables such as smoking habit, sleep duration (hours per day) and physical activity were also recorded. Leisure-time physical activity was assessed using the short form of the Minnesota Leisure Time Physical Activity Questionnaire validated in Spain [20,21] including questions to collect information about types of physical activity, their frequency (number of days), and duration (min/day). Leisure-time activities were computed by assigning a metabolic equivalent score to each activity, multiplied by the time spent for each activity and summing up all activities. The intensity was assigned based on the compendium of physical activity [22]. Finally, personal history of chronic diseases (hypertension, dyslipidemia, and type 2 diabetes) was collected from the participants’ medical records [14].

### Statistical analysis

For this study, we used the latest PREDIMED-Plus baseline database, which was available in June 2020. Multivariable logistic regression models were used in order to assess the relationship between the REGICOR calculated CVR (and its components: sex (male/female), smoking habit (smoker/non-smoker), diabetes status (diabetes/no diabetes), level of total cholesterol (<160, 160–199, 200–239, 240–279 and >280 mg/dL), systolic blood pressure (<120, 120–129, 130–139, 140–159 and >160 mmHg), and diastolic blood pressure (<80, 80–84, 85–89, 90–99 and >100 mmHg)), with the prevalence of depressive status (qualitative dichotomic variable: depressive, when BDI-II score ≥18; non-depressive when BDI-II score <18) at baseline. Odds Ratios (OR) and their 95% Confidence Intervals (CI) were calculated, considering LR as the reference category.

Multivariable linear regression models were used to investigate the associations between the the REGICOR calculated CVR (and its components) with BDI-II scores and with their 2-year changes (quantitative variables). Means, β-coefficients and their 95% CI were calculated.

To control for potential confounding factors, the results were adjusted for the several sociodemographic and lifestyle variables mentioned previously, including the allocation group and center of recruitment at a significance level of p<0.05.

Significance level was established at 0.05 for all analyses. Data were analyzed using Stata (15.0, StataCorp LP, Tx. USA).

## Results

### Participant characteristics at baseline according to CVR

Baseline characteristics of the 6545 participants according to CVR categories are presented in Table 1.

**Table 1:** Baseline characteristics of the study participants.

CVR^a^	LR n = 2141	MR n = 3467	HR n = 937	*P value*
BDI_II≥18^b^ (%)	12.9	11.6	10.8	*0*.*163*
BDI-II (mean ± SD)	8.6 ± 7.6	8.4 ± 7.4	8.3 ± 7.5	*0*.*353*
Sex, women (%)	58.9	47.7	27.6	*<0*.*001*
Age y, (mean ± SD)	65.2 ± 4.9	65.6 ± 4.9	66.6 ± 4.8	*<0*.*001*
BMI (mean ± SD)	32.4 ± 3.4	32.6 ± 3.5	32.6 ± 3.4	*0*.*057*
*<30 (%)*	28.3	25.4	24.8	*0*.*096*
*≥30*, *<35 (%)*	48.4	49.5	49.6	
*≥35 (%)*	23.2	25.2	25.6	
WHR (mean ± SD)	0.97 ± 0.08	0.98 ± 0.08	1.01 ± 0.07	*<0*.*001*
*Above recommended*^c^	91.4	93.1	95.2	*0*.*001*
Physical activity (%)				
*Less active*	64.0	63.8	70.4	*0*.*070*
*Moderately active*	16.5	17.4	11.3	
*Active*	19.5	18.8	18.3	
Educational level (%)				
*Tertiary school*	23.2	21.3	20.6	*0*.*011*
*Secondary school*	26.3	29.7	32.0	
*Primary school*	50.5	49.1	47.4	
Marital status (%)				
*Married*	74.9	76.3	82.7	*<0*.*001*
*Widowed*	10.9	10.9	7.1	
*Divorced/separated*	8.1	7.9	6.0	
*Single*	6.0	4.9	4.2	
Living alone (%)	13.1	13.0	7.9	*<0*.*001*
Employment status (%)				
*Active*	22.6	20.3	16.0	*<0*.*001*
*Retired*	50.2	56.4	68.1	
*Housewife*	17.0	14.8	9.4	
*Others*	10.2	8.5	6.5	
Sleep, h (mean ± SD)	6.7 ± 1.25	7.0 ± 1.22	7.2 ± 1.25	*<0*.*001*
*7–8 h/night (%)*	55.7	59.1	59.7	*0*.*008*
*Less than 7 h/night (%)*	35.4	31.84	29.6	
*More than 8 h/night (%)*	9.0	9.0	10.7	

Values are given as mean ± SD for continuous variables and as percentages for categorical variables. ANOVA test was performed for continuous variables and Pearson’s chi-square test for categorical variables.

^a^ Cardiovascular risk calculated by using REGICOR score: <5% (Low, LR), 5 to 9% (Moderate, MR), ≥10% (High and very high, HR) risk of suffering of a cardiovascular event in 10 years’ time.

^b^ Beck Depression Inventory-II (BDI-II). The participant is considered to suffer of depression when getting a score of 18 or higher.

^c^ For men, values of 0.95 or higher; for women, values of 0.85 or higher

Abbreviations: CVR, cardiovascular risk; BMI, body mass index (kg/m^2^); WHR, waist hip ratio.

The percentages of participants with LR, MR and HR CVR were 32.7%, 53.0% and 14.3% respectively. The percentage of women was inversely associated with CVR. More than 90% of the participants in all CVR categories presented a waist-hip ratio (WHR) higher than recommended (WHR≥ 0.95 for men and WHR≥0.85 for women), increasing with CVR. Most participants were classified as being “less active” across CVR risk groups, with less than 20% of participants reporting being active, independent of their CVR. Around 50% of the participants in each CVR category had received only primary education. Most of the participants (more than 70% in all CVR categories) were married, and this percentage increased with increasing CVR (from 74.9% to 82.7% for LR and HR respectively). For all CVR categories, more than 50% of the participants were retired, increasing the percentage with CVR (from 50.2% in LR to 68.1% in HR). Sleeping hours were the highest for HR participants.

### Cross-sectional associations between CVR components and depressive status

The associations between the individual components of CVR (sex, smoking habit, diabetes status, level of total cholesterol, systolic blood pressure, and diastolic blood pressure), and depressive status (depressive if BDI-II ≥18) are shown in Table 2. Sex (women), smoking and the presence of diabetes were directly associated with depression [OR (95% CI) = 2.86 (2.33, 3.50), 1.38 (1.09, 1.76) and 1.39 (1.17, 1.66), respectively].

**Table 2:** Association between CVR components* and depressive status (BDI-II ≥18) in the PREDIMED-PLUS trial.

CVR components	Model 1	*P value*	Model 2	*P value*
**Sex**				
*Men*	1 (Ref.)		-	
*Women*	**2.86 (2.33, 3.50)**	*<0*.*001*	-	
**Smoking**				
*Non-smokers*	1 (Ref.)		-	
*Smokers*	**1.38 (1.09, 1.76)**	*0*.*008*	-	
**Diabetes**				
*Non diabetes*	1 (Ref.)		-	
*Diabetes*	**1.39 (1.17, 1.66)**	*<0*.*001*	-	
**Cholesterol (mg/dl)**				
*<160*	1 (Ref.)		1 (Ref.)	
*160–199*	0.89 (0.70, 1.14)	*0*.*369*	0.88 (0.68, 1.13)	*0*.*300*
*200–239*	1.05 (0.81, 1.37)	*0*.*702*	1.01 (0.77, 1.12)	*0*.*950*
*240–279*	0.78 (0.55, 1.10)	*0*.*154*	0.78 (0.54, 1.12)	*0*.*544*
*≥280*	1.31 (0.80, 2.16)	*0*.*285*	1.32 (0.79, 2.20)	*0*.*788*
**HDL-Cholesterol (mg/dl)**				
*60–130*	1 (Ref.)		1 (Ref.)	
*35–59*	1.19 (0.89, 1.49)	*0*.*113*	1.21 (0.97, 1.50)	*0*.*098*
*1–34*	1.26 (0.87, 1.81)	*0*.*227*	1.26 (0.87, 1.83)	*0*.*225*
**Systolic blood pressure (mmHg)**				
*<120*	1 (Ref.)		1 (Ref.)	
*120–129*	0.82 (0.62, 1.09)	*0*.*177*	0.84 (0.63, 1.12)	*0*.*228*
*130–139*	0.83 (0.63, 1.09)	*0*.*173*	0.85 (0.64, 1.12)	*0*.*640*
*140–159*	0.76 (0. 57, 1.01)	*0*.*055*	0.78 (0.58, 1.03)	*0*.*583*
*≥160*	0.74 (0.52, 1.07)	*0*.*111*	0.73 (0.51, 1.06)	*0*.*506*
**Diastolic blood pressure (mmHg)**				
*<80*	1 (Ref.)		1 (Ref.)	
*80–84*	1.19 (0.96, 1.48)	*0*.*117*	1.19 (0.96, 1.48)	*0*.*120*
*85–89*	**1.33 (1.03, 1.70)**	***0*.*028***	**1.32 (1.02, 1.70)**	***0*.*033***
*90–99*	0.96 (0.72, 1.30)	*0*.*716*	0.98 (0.73, 1.33)	*0*.*912*
*≥100*	1.50 (0.90, 2.51)	*0*.*901*	1.47 (0.87, 2.48)	*0*.*148*

* According to the Framingham-based REGICOR CVR function. Results are presented as OR and 95% CI for prevalence of depression (BDI-II>18). Model 1: Adjusted for CVR components, recruitment center, marital status, educational level, employment status and sleeping hours. Model 2: (total and HDL cholesterol variables): Additionally, adjusted for statin treatment for variables. Model 2 (systolic and diastolic blood pressure variables): Additionally, adjusted for antihypertensive therapy.

### Cross-sectional associations between CVR and depressive status

The associations between cardiovascular risk levels (LR, MR and HR) and depressive status (depressive if BDI-II ≥18), stratified by CVR factors is presented in Table 3(a). No associations were found for CVR levels and depressive status. However, when CVR was analyzed according to the distribution of each CVR component (sex, smoking habit, diabetes, level of total cholesterol, systolic and diastolic blood pressure), CVR level was directly associated with depressive status in women at HR compared to LR [OR (95% CI) = 1.78 (1.26, 2.50)] at baseline. MR and HR participants with low levels of total cholesterol (<160 mg/mL) showed higher odds of depression compared to LR [OR (95% CI) = 1.77 (1.13, 2.77) and 2.83 (1.25, 6.42) respectively]. On the contrary, MR participants presenting cholesterol levels of 240–279 mg/mL showed lower odds of depressive status compared to LR [OR (95% CI) = 0.50 (0.28, 0.93)], being even lower in MR and HR participants with higher cholesterol levels (≥280 mg/mL) [OR (95% CI) = 0.26 (0.07, 0.98) and 0.23 (0.05, 0.95) respectively].

**Table 3:** Association between cardiovascular risk and a) depressive status (BDI-II ≥18) and b) BDI-II score, in the PREDIMED-PLUS trial, stratified by CVR factors, at baseline.

CVR^a^	a) CARDIOVASCULAR RISK AND DEPRESSIVE STATUS			b) CVR AND BDI-II SCORE		
	[OR (95% CI)]			[Mean±SE, β-coef. (95% CI)]		
	LR	MR	HR	LR	MR	HR
**All participants**	1 (Ref.)	0.97 (0.82, 1.15)	1.02 (0.79, 1.31)	8.45±0.16	8.46±0.12	8.74±0.24
				0 (Ref.)	0.01 (-0.38, 0.41)	0.28 (-0.03, 0.86)
*P value*		*0*.*715*	*0*.*901*		*0*.*945*	*0*.*309*
**Men**	1 (Ref.)	0.86 (0.62, 1.18)	0.87 (0.57, 1.32)	6.83±0.21	6.68±0.15	7.24±0.25
				0 (Ref.)	-0.15 (-0.67, 0.36)	0.41 (-0.24, 1.05)
*P value*		*0*.*346*	*0*.*515*		*0*.*561*	*0*.*219*
**Women**	1 (Ref.)	1.14 (0.92, 1.39)	**1.78 (1.26, 2.50)**	9.75±0.22	10.4±0.19	11.8±0.49
				0 (Ref.)	**0.67 (0.08, 1.26)**	**2.02 (0.95, 3.09)**
*P value*		*0*.*226*	*0*.*001*		*0*.*027*	*<0*.*001*
**Non-smokers**	1 (Ref.)	0.93 (0.78, 1.11)	1.11 (0.84, 1.48)	8.44±0.16	8.42±0.13	8.79±0.29
				0 (Ref.)	-0.02 (-0.43, 0.39)	0.35 (-0.30, 1.00)
*P value*		*0*.*431*	*0*.*464*		*0*.*931*	*0*.*295*
**Smokers**	1 (Ref.)	1.20 (0.61, 2.34)	0.71 (0.34, 1.50)	9.08±0.76	8.86±0.37	8.40±0.44
				0 (Ref.)	-0.22 (-1.88, 1.45)	-0.68 (-2.44, 1.09)
*P value*		*0*.*596*	*0*.*372*		*0*.*795*	*0*.*4512*
**Non diabetes**	1 (Ref.)	0.89 (0.73, 1.09)	**0.59 (0.37, 0.93)**	8.39±0.17	8.12±0.14	7.97±0.37
				0 (Ref.)	-0.28 (-0.72, 0.16)	-0.43 (-1.24, 0.38)
*P value*		*0*.*260*	*0*.*023*		*0*.*213*	*0*.*300*
**Diabetes**	1 (Ref.)	1.07 (0.75, 1.53)	1.26 (0.84, 1.88)	8.80±0.39	9.19±0.24	9.30±0.32
				0 (Ref.)	0.39 (-0.50, 1.29)	0.50 (-0.50, 1.49)
*P value*		*0*.*718*	*0*.*258*		*0*.*388*	*0*.*329*
**Total colesterol (mg/dl)**						
*<160*	1 (Ref.)	**1.77 (1.13, 2.77)**	**2.83 (1.25, 6.42)**	7.75±0.30	8.95±0.38	9.68±0.96
				0 (Ref.)	**1.19 (0.23, 2.16)**	1.93 (-0.05, 3.90)
*P value*		*0*.*012*	*0*.*012*		*0*.*015*	*0*.*057*
*160–199*	1 (Ref.)	0.88 (0.65, 1.18)	1.05 (0.70, 1.60)	8.27±0.25	8.02±0.19	8.47±0.36
				0 (Ref.)	-0.25 (-0.88, 0.37)	0.20 (-0.68, 1.08)
*P value*		*0*.*395*	*0*.*801*		*0*.*427*	*0*.*659*
*200–239*	1 (Ref.)	0.96 (0.70, 1.30)	0.66 (0.41, 1.08)	8.88±0.33	8.86±0.2	8.70±0.43
				0 (Ref.)	0.01 (-0.78, 0.76)	-0.18 (-0.25, 0.89)
*P value*		*0*.*780*	*0*.*098*		*0*.*974*	*0*.*743*
*240–279*	1 (Ref.)	**0.50 (0.28, 0.93)**	0.98 (0.46, 2.08)	8.87±0.53	8.18±0.39	9.44±0.65
				0 (Ref.)	-0.69 (-2.00, 0.62)	0.57 (-1.10, 2.25)
*P value*		*0*.*028*	*0*.*955*		*0*.*300*	*0*.*501*
*≥280*	1 (Ref.)	**0.26 (0.07, 0.98)**	**0.23 (0.05, 0.95)**	11.34±1.33	10.54±1.01	8.43±1.21
				0 (Ref.)	-0.80 (-4.15, 2.53)	-2.91 (-6.61, 0.79)
*P value*		*0*.*047*	*0*.*042*		*0*.*634*	*0*.*112*

Results are presented as a) OR and 95% CI for prevalence of depression (Beck-II score of 18 or higher) and b) adjusted means±SE and β-coefficients and 95% CI for Beck-II score as continuous variable, according to CVR according to CVR (LR, n = 2141; MR, n = 3467; HR, n = 937). LR as the reference category. Adjusted for recruitment canter, marital status, educational level, employment status and sleeping hours.

^a^ Cardiovascular risk calculated by REGICOR score: <5% (Low, LR), 5 to 9% (Moderate, MR), ≥10% (High and very high, HR) risk of suffering of a cardiovascular event in 10 years’ time.

### Cross-sectional associations between CVR and BDI-II score

The associations between CVR and BDI-II score stratified by CVR factors is presented in Table 3(b). HR and MR women presented higher means of BDI-II score compared to LR women (adjusted mean±SE = 10.4±0.19, 11.8±0.49 and 9.75±0.22 respectively). MR participants with low levels of total cholesterol (<160 mg/mL) also showed higher means of BDI-II score compared to LR with the same level of total cholesterol (adjusted mean±SE = 8.95±0.38 and 7.75±0.30 respectively).

### Longitudinal associations between CVR at baseline and changes in BDI-II score after 2 years of follow-up

No associations were found between CVR and changes in BDI-II score (BDI-II score after 2 years, minus BDI-II score at baseline) after 2 years of follow up (Table 4). However, when the results were stratified according to CVR components, some statistically significant associations were found. All individuals with diabetes lowered their BDI-II score after 2 years of follow up, being this decrease lower in MR and HR than in LR diabetic individuals (adjusted mean±SE = -0.52±0.20, -0.41±0.27 and -1.25±0.31 respectively). Moreover, HR participants with low levels of HDL-cholesterol levels (<35 mg/mL) showed a smaller decrease in BDI-II score compared to LR participants (mean±SE = -0.56±0.33 and -2.00±0.50 respectively). On the contrary, MR and HR participants presenting high total cholesterol levels (240–279 mg/mL) decreased their BDI-II score after 2 years of follow up, compared to LR participants, which did not decrease it (adjusted mean±SE = -0.83±0.37, -0.77±0.64 and 0.97±0.52 respectively).

**Table 4:** Longitudinal associations between baseline CVR and 2 years changes in BDI-II score in participants with a BDI-II score <18 in the PREDIMED-PLUS trial at baseline.

CVR^a^			LR	MR	HR
**All participants**	Mean change±SE		-0.86±0.13	-0.76±0.10	-0.64±0.20
	β-coef. (95% CI)		0 (Ref.)	0.10 (-0.23, 0.42)	0.22 (-0.25, 0.68)
	*P value*			*0*.*560*	*0*.*366*
**Men**	Mean change±SE		-1.18±0.17	-0.87±0.12	-0.77±0.20
	β-coef. (95% CI)		0 (Ref.)	0.31 (-0.09, 0.71)	0.41 (-0.10, 0.92)
	*P value*			*0*.*130*	*0*.*115*
**Women**	Mean change±SE		-0.69±0.20	-0.62±0.17	0.18±0.48
	β-coef. (95% CI)		0 (Ref.)	0.08 (-0.44, 0.59)	0.87 (-0.14, 1.88)
	*P value*			*0*.*768*	*0*.*093*
**Non-smokers**	Mean change±SE		-0.84±0.13	-0.74±0.11	-0.52±0.24
	β-coef. (95% CI)		0 (Ref.)	0.10 (-0.23, 0.43)	0.32 (-0.23, 0.86)
	*P value*			*0*.*564*	*0*.*254*
**Smokers**	Mean change±SE		-0.93±0.65	-0.87±0.31	-1.13±0.36
	β-coef. (95% CI)		0 (Ref.)	0.06 (-1.35, 1.48)	-0.19 (-1.69, 1.30)
	*P value*			*0*.*928*	*0*.*798*
**Non diabetes**	Mean change±SE		-0.78±0.14	-0.85±0.12	-1.03±0.31
	β-coef. (95% CI)		0 (Ref.)	-0.07 (-0.43, 0.29)	-0.26 (-0.93, 0.41)
	*P value*			*0*.*695*	*0*.*454*
**Diabetes**	Mean change±SE		-1.25±0.31	-0.52±0.20	-0.41±0.27
	β-coef. (95% CI)		0 (Ref.)	**0.73 (0.01, 1.44)**	**0.84 (0.03, 1.65)**
	*P value*			*0*.*048*	*0*.*042*
**HDL-cholesterol (mg/dl)**					
**60–130**	Mean change±SE		-0.45±0.22	-0.71±0.66	0.38±1.50
	β-coef. (95% CI)		0 (Ref.)	-0.25 (-1.63, 1.12)	0.83 (-2.15, 3.81)
	*P value*			*0*.*718*	*0*.*584*
**35–59**	Mean change±SE		-0.96±0.17	-0.72±0.10	-0.70±0.24
	β-coef. (95% CI)		0 (Ref.)	0.24 (-0.16, 0.64)	0.27 (-0.32, 0.85)
	*P value*			*0*.*237*	*0*.*369*
**1–34**	Mean change±SE		-2.00±0.50	-1.59±0.32	-0.56±0.33
	β-coef. (95% CI)		0 (Ref.)	0.41 (-0.76, 1.58)	**1.44 (0.23, 2.65)**
	*P value*			*0*.*490*	*0*.*020*
**Total cholesterol (mg/dl)**					
**<160**	Mean change±SD		-1.19±0.21	-0.35±0.29	-1.67±0.75
	β-coef. (95% CI)		0 (Ref.)	**0.84 (0.12, 1.56)**	-0.48 (-2.02, 1.06)
	*P value*			*0*.*023*	*0*.*541*
**160–199**	Mean change±SD		-0.75±0.20	-0.84±0.15	-0.53±0.30
	β-coef. (95% CI)		0 (Ref.)	-0.09 (-0.60, 0.41)	0.23 (-0.49, 0.94)
	*P value*			*0*.*721*	*0*.*537*
**200–239**	Mean change±SD		-0.17±0.30	-0.82±0.17	-0.68±0.36
	β-coef. (95% CI)		0 (Ref.)	0.35 (-0.29, 0.99)	0.49 (-0.41, 1.40)
	*P value*			*0*.*282*	*0*.*288*
**240–279**	Mean change±SD		0.97±0.52	-0.83±0.37	-0.77±0.64
	β-coef. (95% CI)		0 (Ref.)	**-1.80 (-3.06, -0.53)**	**-1.74 (-3.39, -0.10)**
	*P value*			*0*.*006*	*0*.*038*
**≥280**	Mean change±SD		1.17±1.81	-1.85±1.14	0.12±1.49
	β-coef. (95% CI)		0 (Ref.)	-3.02 (-7.57, 1.53)	-1.05 (-6.28, 4.18)
	*P value*			0.188	0.688

Results are presented as adjusted means±SE, together with β-coefficients and 95% CI with LR as the reference category (0), for 2-year changes in depression symptomatology (BDI-II after 2 years of follow-up minus BDI-II score at baseline), according to CVR (LR, n = 1497; MR, n = 2436; HR, n = 633). Adjusted by BDI-II score at baseline, recruitment center, intervention group, marital status, educational level, employment status and sleeping hours.

^a^ Cardiovascular risk calculated by REGICOR score: <5% (Low, LR), 5 to 9% (Moderate, MR), ≥10% (High and very high, HR) risk of suffering of a cardiovascular event in 10 years’ time

When all participants, independently of their baseline depressive status, were included in the analysis (S1 Table), the magnitude of the changes in BDI-II score observed after 2 years of follow up was higher, with a significant difference in depressive score reduction between HR and LR women (adjusted mean±SE = -0.51±0.47 and -1.89±0.20 respectively), HR vs. LR participants with diabetes (adjusted mean±SE = -1.24±0.28 and -2.05±0.65 respectively) and among HR vs. LR participants with very low HDL-cholesterol levels (<35 mg/dl) (adjusted mean±SE = -1.02±0.35 and 2.68±0.51 respectively).

Results from the analysis of the BDI-II changes stratified by intervention group (an intensive weight loss lifestyle intervention based on an energy-restricted Mediterranean diet, physical activity promotion, and behavioral support or a control group) and sex are presented in Supporting information (S2 Table). All participants decreased their BDI-II score after 2 years of follow up. However, the association between CVR and these changes were modified by the intervention group (p-interaction = 0.002). Participants in the control group showed an inverse relationship between CVR and the decrease in BDI-II score (-0.73±0.18, -0.49±0.14 and -0.07±0.29 for LR, MR and HR respectively), whereas participants in the intervention group showed a more direct relationship with CVR with those participants at HR showing the highest decrease in depressive symptoms after 2-years (-0.97±0.18, -1.07±0.14 and -1.26±0.27 for LR, MR and HR respectively). Furthermore, the decreases in BDI-II score were of a higher magnitude in the intervention group compared to the control group. The same trend was observed in men after stratification by sex. When only men were considered in the analysis, an effect modification of dietary intervention in the association between CVR and depressive symptoms reduction was detected (p-interaction CVR and intervention group <0.005). On the other hand, no interaction between CVR group and intervention group was observed in women. Both intervention and control groups showed an inverse relationship between CVR and the decrease in BDI-II score in women, with this decrease being higher in women assigned to the intervention group. When all participants were included in the analysis (S3 Table), the magnitude of the changes in BDI-II score observed after 2 years of follow-up was higher.

## Discussion

The present analysis was carried out within the PREDIMED-Plus trial as an observational prospective cohort study, where participants classified at baseline as low, medium, and high/very high CVR were followed for 2 years. Among women and participants with low levels of baseline total cholesterol (<160 mg/mL), CVR was directly associated with depression. On the contrary, among participants with elevated levels of total cholesterol CVR at baseline showed an inverse association with the presence of depressive symptomatology. In the longitudinal analysis, after two years of follow-up, BDI-II score decreased for all participants regardless of the CVR level. However, participants at MR and/or HR presented smaller decreases in BDI-II score after 2 years of follow up compared to LR participants when they presented diabetes or low levels of HDL-cholesterol. In general, differences in BDI-II score after 2 years of follow up were small, probably because we investigated participants without depression (with BDI-II score lower than 18). In fact, when all participants were included in the analysis, the magnitude of the changes in BDI-II score observed after 2 years of follow up was higher. Other studies have also suggested that a higher load of cardiovascular risk factors without the presence of CVD may imply a higher risk of depression. Thus, a higher predicted CVD risk was strongly associated with a higher future incidence of depression, both in younger and older adults un the SUN cohort [11]. Also, results from the cross-sectional and a longitudinal analysis of the ELSA-Brazil study, where healthy adults were classified into poor, intermediate, and optimal cardiovascular health [23], showed that poor cardiovascular health is associated with depression [10], and triples the risk of depression after almost 4 years of follow up [9] in otherwise healthy adults. Furthermore, these effects were more pronounced in women and in participants younger than 55 years old. Altogether, the literature suggests cardiovascular metrics are associated with risk of depression. Based on the associations observed in the current findings and the literature, it is coherent to think that CVD and depression may share either common pathophysiological mechanisms or risk factors [24,25] or both.

Therefore, ameliorating cardiovascular health, by acting on the common pathophysiological mechanisms and/or on the CVR factors, could decrease depression risk development. Although the mechanisms explaining the link between CVD and depression are complex and remain unclear, obesity, MetS, reduced insulin sensitivity, elevations in plasma homocysteine levels, endothelial dysfunction, increased production of pro-inflammatory cytokines or a single-nucleotide polymorphism in the BDNF gene (BDNFVal66Met) are plausible candidates [11,26]. Very recently, the proprotein convertase subtilisin/kexin type 9 (PCSK9), has been added to these candidates [27]. Of these, inflammation involving the immune system is thought to be an important common mechanism of depression and heart disease [28,29], with specific inflammatory cytokines or pathways being potential targets for the prevention and treatment of the concurrent diseases [3]. In this regard, a limitation of the current analyses is that data on inflammatory markers were not available in our study.

By finding shared molecular mechanisms, we can use tools for the prevention of both conditions, such as the adoption of an anti-inflammatory dietary pattern together with a healthy lifestyle to decrease inflammation may be used. In this sense, existing literature indicates that food patterns with a high content in fruit and vegetables, olive oil, tree nuts, fish and whole grains, and low in meats, meat products, commercial bakery products, trans fats, sugary desserts and sugar-sweetened beverages, such as the Mediterranean Diet, are associated with a reduced risk of depression [30] and CVD [31]. In addition, to the anti-inflammatory effects of the Mediterranean Diet, the effect that this diet exerts on depression has been linked to the effects on gut microbiota through the gut microbiota-brain-axes [32]. There are many reasons to support the biological plausibility that cardioprotective food patterns are also protective against major depression, explained elsewhere [30]. In this sense, although both, intervention, and control groups, showed a decrease in BDI-II score after 2 years of follow-up, the difference was of higher magnitude for participants assigned to the intervention group and at high risk of cardiovascular disease, especially among men. This suggests that the intervention is more effective on depressive symptoms in those participants at higher CVR and would likely be less effective on those with low risk factors.

Regarding shared cardiovascular and depression risk factors, we observed that the frequency of having a BDI-II score equal to or higher than 18 varied depending on sex, being higher in women than in men. Similarly to our results, it has been stated that women have two-times the probability of suffering from depression than men, across a variety of nations, cultures and ethnicities [5]. In addition, we found that this association was higher in women at medium or high cardiovascular risk. Women also appear to be more strongly affected by psychosocial stressors related to CVD and depression, including alterations in the hypothalamic–pituitary–adrenal axis functioning and autonomic nervous system, as well as by direct and indirect effects of chronic stress compared to men [33].

Although we did not find an association between total cholesterol levels and depression (BDI-II score equal or higher than 18, we found that this variable could influence the association of cardio-vascular risk with depression. Cholesterol is a core component of the central nervous system, essential for the cell membrane stability and the correct functioning of neurotransmission [34]. It has been suggested that serum cholesterol levels may be positively associated with serotonergic receptor function [35]. Kaplan and colleagues proposed the cholesterol-serotonin hypothesis after the finding of an inverse relationship between levels of serum cholesterol and aggressive behavior [36]. This hypothesis suggests that low dietary cholesterol intake leads to decreased central serotonergic activity. Low HDL-cholesterol has been associated with increased odds of new- onset depressive symptoms in participants aged 65–70 years [13] and it has been suggested that serum cholesterol levels [37], especially LDL-cholesterol [38,39], are inversely correlated with depressive mood. It has been established that persons with type 2 diabetes have both increased prevalence and incidence of depression relative to persons without diabetes [40]. The pathophysiological mechanisms that could explain this association include the psychological burden of being ill which may play an important role in triggering anxiety and depression, structural changes in the brain or the use of anti-depressive treatment [41].

Some potential limitations of our study need to be mentioned. Our participants were aged between 55 and 75 years, were overweight or obese, and all met criteria for metabolic syndrome. Nevertheless, the lack of representativeness does not preclude the establishment of associations [42]. Moreover, the follow-up time (2 years) could have been not enough to detect changes of higher magnitude in depressive symptoms. In addition, data on co-medications and specific drugs for depression or anxiety were not available. Finally, although all the results were adjusted for a variety of major potential confounders, we cannot exclude the presence of some unknown or unmeasured factors that could partly explain the reported results.

Strengths of the study are the large sample size, the population with consists of people of both sexes, the adjustment for a wide array of potential confounders, the use of validated tools to assess information and the ability to conduct both cross-sectional and longitudinal analyses.

The cross-sectional nature of the analyses carried out allowed us to detect an association between CVR and depression, but as Bianco et al point out [43], the dilemma of the sense of causality cannot be resolved. In this case, our hypothesis is that both problems share a common enteropathogenic basis, which would improve with improved diet and lifestyles; however, it has not been possible to demonstrate that this occurs in the two years following the beginning of the intervention.

In conclusion, high and very high cardiovascular risk was associated with depressive symptoms, especially in women. Moreover, the role of other factors such as total and HDL cholesterol or the adherence to the Mediterranean diet in the association with cardiovascular risk and depression deserves further research. Identification of CVR factors as early indicators of depression development in older adults with overweight or obesity and metabolic syndrome should be performed. Improving cardiovascular health could contribute to the prevention of the onset of depression in this group of population.

## Supporting information

S1 Table Longitudinal associations between baseline CVR and its components, and 2 years changes in BDI-II score in the PREDIMED-Plus trial. Results are presented as adjusted means±SE, together with β-coefficients and 95% CI with LR as the reference category (0), for 2-year changes in depression symptomatology (BDI-II after 2 years of follow-up minus BDI-II score at baseline), according to CVR (LR, n = 1714; MR, n = 2742; HR, n = 707). Adjusted by BDI-II score at baseline, recruitment center, intervention group, marital status, educational level, employment status and sleeping hours. ^a^Cardiovascular risk calculated by REGICOR score: <5% (Low, LR), 5 to 9% (Moderate, MR), ≥10% (High and very high, HR) risk of suffering of a cardiovascular event in 10 years’ time.
(DOCX)

S2 Table Longitudinal associations between baseline CVR and 2 years changes in BDI-II score in participants with a BDI-II score <18 in the PREDIMED-PLUS trial at baseline, stratified by intervention group and sex. Results are presented as adjusted means±SE, together with β-coefficients and 95% CI with LR as the reference category (0), for 2-year changes in depression symptomatology (BDI-II after 2 years of follow-up minus BDI-II score at baseline), according to CVR (LR, n = 1497; MR, n = 2436; HR, n = 633), stratified by intervention group and sex. Adjusted by BDI-II score at baseline, recruitment center, marital status, educational level, employment status and sleeping hours. ^a^Cardiovascular risk calculated by REGICOR score: <5% (Low, LR), 5 to 9% (Moderate, MR), ≥10% (High and very high, HR) risk of suffering of a cardiovascular event in 10 years’ time.
(DOCX)

S3 Table Longitudinal associations between baseline CVR and 2 years changes in BDI-II score in the PREDIMED-PLUS trial, stratified by intervention group and sex. Results are presented as adjusted means±SE, together with β-coefficients and 95% CI with LR as the reference category (0), for 2-year changes in depression symptomatology (BDI-II after 2 years of follow-up minus BDI-II score at baseline), according to CVR (LR, n = 1714; MR, n = 2742; HR, n = 707). Stratified by intervention group and sex. Adjusted by BDI-II score at baseline, recruitment center, marital status, educational level, employment status and sleeping hours. ^a^Cardiovascular risk calculated by REGICOR score: <5% (Low, LR), 5 to 9% (Moderate, MR), ≥10% (High and very high, HR) risk of suffering of a cardiovascular event in 10 years’ time.
(DOCX)
