# Supplementary material for: Contribution of cardio-vascular risk factors to depressive status in the PREDIMED-PLUS Trial. A cross-sectional and a 2-year longitudinal study
Source: PLoS One. 2022 Apr 13;17(4):e0265079. doi: 10.1371/journal.pone.0265079 (PMC9007355; doi:10.1371/journal.pone.0265079)
Supplement: S1 Table — Results are presented as adjusted means±SE, together with β-coefficients and 95% CI with LR as the reference category (0), for 2-year changes in depression symptomatology (BDI-II after 2 years of follow-up minus BDI-II score at baseline), according to CVR (LR, n = 1714; MR, n = 2742; HR, n = 707). Adjusted by BDI-II score at baseline, recruitment center, intervention group, marital status, educational level, employment status and sleeping hours. aCardiovascular risk calculated by REGICOR score: <5% (Low, LR), 5 to 9% (Moderate, MR), ≥10% (High and very high, HR) risk of suffering of a cardiovascular event in 10 years’ time. (DOCX) [file pone.0265079.s001.docx]

**S1 Table.** **Longitudinal associations between baseline CVR and its components, and 2 years changes in BDI-II score in the PREDIMED-Plus trial.**

| **CVR^a^** | **Low** | **Moderate** | **High/very high** |
| --- | --- | --- | --- |
| **All participants** |  |  |  |
| Mean change±SE | -1.70±0.14 | -1.72±0.11 | -1.34±0.21 |
| β-coef. (95% CI) | 0 (Ref.) | -0.01 (-0.35, 0.33) | 0.37 (-0.13, 0.86) |
| **Men** |  |  |  |
| Mean change±SE | -1.77±0.17 | -1.45±0.12 | -1.31±0.21 |
| β-coef. (95% CI) | 0 (Ref.) | 0.32 (-0.10, 0.74) | 0.46 (-0.08, 1.00) |
| **Women** |  |  |  |
| Mean change±SE | -1.89±0.20 | -2.02±0.17 | -0.51±0.47 |
| β-coef. (95% CI) | 0 (Ref.) | -0.21 (-0.74, 0.32) | **1.29 (0.28, 2.31)** |
| **Non-smokers** |  |  |  |
| Mean change±SE | -1.68±0.14 | -1.66±0.11 | -1.15±0.26 |
| β-coef. (95% CI) | 0 (Ref.) | 0.02 (-0.33, 0.37) | 0.53 (-0.05, 1.10) |
| **Smokers** |  |  |  |
| Mean change±SE | -2.05±0.65 | -2.01±0.31 | -1.89±0.38 |
| β-coef. (95% CI) | 0 (Ref.) | 0.04, (-1.39, 1.46) | 0.16 (-1.35, 1.67) |
| **Non diabetes** |  |  |  |
| Mean change±SE | -1.54±0.15 | -1.73±0.12 | -1.87±0.34 |
| β-coef. (95% CI) | 0 (Ref.) | -0.20 (-0.58, 0.14) | -0.34 (-1.07, 0.39) |
| **Diabetes** |  |  |  |
| Mean change±SE | -2.21±0.32 | -1.63±0.20 | -1.24±0.28 |
| β-coef. (95% CI) | 0 (Ref.) | 0.58 (-0.17, 1.34) | **0.98 (0.13, 1.82)** |
| **Total cholesterol** | | | |
| ***<160*** |  |  |  |
| Mean change±SE | -2.17±0.23 | -1.63±0.30 | -2.68±0.79 |
| β-coef. (95% CI) | 0 (Ref.) | 0.55 (-0.22, 1.31) | -0.51 (-2.14, 1.12) |
| ***160-199*** |  |  |  |
| Mean change±SE | -1.50±0.21 | -1.62±0.16 | -1.06±0.30 |
| β-coef. (95% CI) | 0 (Ref.) | -0.12 (-0.60, 0.39) | 0.43 (-0.30, 1.16) |
| ***200-239*** |  |  |  |
| Mean change±SE | -2.03±0.30 | -1.88±0.18 | -1.40±0.40 |
| β-coef. (95% CI) | 0 (Ref.) | 0.14 (-0.55, 0.83) | 0.62 (-0.37, 1.62) |
| ***240-279*** |  |  |  |
| Mean change±SE | 0.40±0.51 | -1.71±0.38 | 1.43±0.64 |
| β-coef. (95% CI) |  | **-2.11 (-3.40, -0.82)** | **-1.83 (-3.49, -0.18)** |
| ***≥280*** |  |  |  |
| Mean change±SE | -1.18±1.55 | -3.84±1.03 | 0.28±1.44 |
| β-coef. (95% CI) |  | -2.66 (-6.58, 1.26) | 1.46 (-3.35, 6.27) |
| **HDL-cholesterol** | | | |
| ***60-130*** |  |  |  |
| Mean change±SE | -1.57±0.23 | -2.43±0.70 | -0.83±1.66 |
| β-coef. (95% CI) |  | -0.86 (-2.30, 0.59) | 0.75 (-2.54, 4.03) |
| ***35-59*** |  |  |  |
| Mean change±SE | -1.74±0.18 | -1.64±0.11 | -1.40±0.25 |
| β-coef. (95% CI) |  | 0.11 (-0.31, 0.53) | 0.34 (-0.27, 0.96) |
| ***1-34*** |  |  |  |
| Mean change±SE | -2.68±0.51 | -2.33±0.33 | -1.02±0.35 |
| β-coef. (95% CI) |  | 0.35 (-0.85, 1.56) | **1.66 (0.41, 2.91)** |

Results are presented as adjusted means±SE, together with β-coefficients and 95% CI with LR as the reference category (0), for 2-year changes in depression symptomatology (BDI-II after 2 years of follow-up minus BDI-II score at baseline), according to CVR (LR, n=1714; MR, n=2742; HR, n=707). Adjusted by BDI-II score at baseline, recruitment center, intervention group, marital status, educational level, employment status and sleeping hours.

^a^ Cardiovascular risk calculated by REGICOR score: <5% (Low, LR), 5 to 9% (Moderate, MR), ≥10% (High and very high, HR) risk of suffering of a cardiovascular event in 10 years’ time.
