# Supplementary material for: Contribution of cardio-vascular risk factors to depressive status in the PREDIMED-PLUS Trial. A cross-sectional and a 2-year longitudinal study
Source: PLoS One. 2022 Apr 13;17(4):e0265079. doi: 10.1371/journal.pone.0265079 (PMC9007355; doi:10.1371/journal.pone.0265079)
Supplement: S2 Table — Results are presented as adjusted means±SE, together with β-coefficients and 95% CI with LR as the reference category (0), for 2-year changes in depression symptomatology (BDI-II after 2 years of follow-up minus BDI-II score at baseline), according to CVR (LR, n = 1497; MR, n = 2436; HR, n = 633), stratified by intervention group and sex. Adjusted by BDI-II score at baseline, recruitment center, marital status, educational level, employment status and sleeping hours. aCardiovascular risk calculated by REGICOR score: <5% (Low, LR), 5 to 9% (Moderate, MR), ≥10% (High and very high, HR) risk of suffering of a cardiovascular event in 10 years’ time. (DOCX) [file pone.0265079.s002.docx]

**S2 Table. Longitudinal associations between baseline CVR and 2 years changes in BDI-II score in participants with a BDI-II score <18 in the PREDIMED-PLUS trial at baseline, stratified by intervention group and sex.**

| **CVR^a^** | **LR** | **MR** | **HR** |
| --- | --- | --- | --- |
| **Control group**  Mean change±SE  β-coef. (95% CI) | -0.73±0.18  0 (Ref.) | -0.49±0.14  0.26 (-0.21, 0.71) | -0.07±0.29  0.66 (-0.02, 1.34) |
| **Men**  Mean change±SE  β-coef. (95% CI) | -1.12±0.24  0 (Ref.) | -0.56±0.17  0.56 (-0.01, 1.14) | -0.18±0.29  **0.94 (0.20, 1.68)** |
| **Women**  Mean change±SE  β-coef. (95% CI) | -0.53±0.28  0 (Ref.) | -0.36±0.24  0.18 (-0.56, 0.91) | 0.72±0.71  1.25 (-0.25, 2.76) |
| **Intervention group**  Mean change±SE  β-coef. (95% CI) | -0.97±0.18  0 (Ref.) | -1.07±0.14  -0.10 (-0.54, 0.35) | -1.26±0.27  -0.28 (-0.93, 0.36) |
| **Men**  Mean change±SE  β-coef. (95% CI) | -1.20±0.23  0 (Ref.) | -1.22±0.16  -0.02 (-0.59, 0.54) | -1.38±0.27  -0.18 (-0.89, 0.53) |
| **Women**  Mean change±SE  β-coef. (95% CI) | -0.84±0.28  0 (Ref.) | -0.89±0.24  -0.05 (-0.78, 0.68) | -0.55±0.64  0.29 (-1.09, 1.68) |

Results are presented as adjusted means±SE, together with β-coefficients and 95% CI with LR as the reference category (0), for 2-year changes in depression symptomatology (BDI-II after 2 years of follow-up minus BDI-II score at baseline), according to CVR (LR, n=1497; MR, n=2436; HR, n=633), stratified by intervention group and sex. Adjusted by BDI-II score at baseline, recruitment center, marital status, educational level, employment status and sleeping hours.

^a^ Cardiovascular risk calculated by REGICOR score: <5% (Low, LR), 5 to 9% (Moderate, MR), ≥10% (High and very high, HR) risk of suffering of a cardiovascular event in 10 years’ time.
