# Supplementary material for: Contribution of cardio-vascular risk factors to depressive status in the PREDIMED-PLUS Trial. A cross-sectional and a 2-year longitudinal study
Source: PLoS One. 2022 Apr 13;17(4):e0265079. doi: 10.1371/journal.pone.0265079 (PMC9007355; doi:10.1371/journal.pone.0265079)
Supplement: S3 Table — Results are presented as adjusted means±SE, together with β-coefficients and 95% CI with LR as the reference category (0), for 2-year changes in depression symptomatology (BDI-II after 2 years of follow-up minus BDI-II score at baseline), according to CVR (LR, n = 1714; MR, n = 2742; HR, n = 707). Stratified by intervention group and sex. Adjusted by BDI-II score at baseline, recruitment center, marital status, educational level, employment status and sleeping hours. aCardiovascular risk calculated by REGICOR score: <5% (Low, LR), 5 to 9% (Moderate, MR), ≥10% (High and very high, HR) risk of suffering of a cardiovascular event in 10 years’ time. (DOCX) [file pone.0265079.s003.docx]

**S3 Table. Longitudinal associations between baseline CVR and 2 years changes in BDI-II score in the PREDIMED-PLUS trial, stratified by intervention group and sex**.

| **CVR^a^** | **LR** | **MR** | **HR** |
| --- | --- | --- | --- |
| **Control Group** |  |  |  |
| Mean change±SE | -1.62±0.19 | -1.38±0.15 | -0.85±0.31 |
| β-coef. (95% CI) | 0 (Ref.) | 0.25 (-0.23, 0.72) | 0.77 (0.06, 1.45) |
| **Men** |  |  |  |
| Mean change±SE | -1.81±0.25 | -1.06±0.18 | -0.67±0.30 |
| β-coef. (95% CI) | 0 (Ref.) | **0.75 (0.14, 1.35)** | **1.14 (0.36, 1.92)** |
| **Women** |  |  |  |
| Mean change±SE | -1.66±0.28 | -1.72±0.24 | -0.35±0.69 |
| β-coef. (95% CI) | 0 (Ref.) | -0.06 (-0.80, 0.68) | 1.31 (-0.17, 2.79) |
| **Intervention Group** |  |  |  |
| Mean change±SE | -1.77±0.19 | -2.10±0.15 | -1.81±0.29 |
| β-coef. (95% CI) | 0 (Ref.) | -0.3 4(-0.82, 0.14) | -0.04 (-0.73, 0.65) |
| **Men** |  |  |  |
| Mean change±SE | -1.71±0.24 | -1.89±0.17 | -1.92±0.28 |
| β-coef. (95% CI) | 0 (Ref.) | -0.18 (-0.77, 0.41) | -0.21 (-0.95, 0.53) |
| **Women** |  |  |  |
| Mean change±SE | -1.97±0.29 | -2.35±0.25 | -0.57±0.66 |
| β-coef. (95% CI) | 0 (Ref.) | -0.38 (-1.14, 0.39) | 1.40 (-0.03, 2.82) |

Results are presented as adjusted means±SE, together with β-coefficients and 95% CI with LR as the reference category (0), for 2-year changes in depression symptomatology (BDI-II after 2 years of follow-up minus BDI-II score at baseline), according to CVR (LR, n=1714; MR, n=2742; HR, n=707). stratified by intervention group and sex. Adjusted by BDI-II score at baseline, recruitment center, marital status, educational level, employment status and sleeping hours.

^a^ Cardiovascular risk calculated by REGICOR score: <5% (Low, LR), 5 to 9% (Moderate, MR), ≥10% (High and very high, HR) risk of suffering of a cardiovascular event in 10 years’ time.
